# Spatial and temporal assessment of soil degradation risk in Europe

**DOI:** 10.1038/s41598-025-33318-7

**Published:** 2025-12-24

**Authors:** Mehdi H. Afshar, Amirhossein Hassani, Milad Aminzadeh, Pasquale Borrelli, Panos Panagos, David A. Robinson, Dani Or, Nima Shokri

**Affiliations:** 1 Institute of Geo-Hydroinformatics, Hamburg University of Technology, Hamburg, Germany; 2 United Nations University Hub on Engineering to Face Climate Change at the Hamburg University of Technology, United Nations University Institute for Water, Environment and Health (UNU-INWEH), Hamburg, Germany; 3 The Climate and Environmental Research Institute NILU, P.O. Box 100, Kjeller, 2027 Norway; 4 Department of Environmental Sciences, University of Basel, Basel, 4056 Switzerland; 5 European Commission, Joint Research Centre (JRC), Ispra, Italy; 6 Centre for Ecology & Hydrology, Bangor, UK; 7 Department of Civil & Environmental Engineering, University of Nevada, Reno, NV USA; 8 Environmental Systems Science, ETH Zurich, Zurich, Switzerland

**Keywords:** Soil monitoring, Machine learning, Land cover, Climate variability, Soil health indicator, Environmental policy, Climate sciences, Ecology, Ecology, Environmental sciences

## Abstract

Soil degradation threatens agricultural productivity and ecosystem resilience across Europe, yet spatially consistent assessments of its intensity and drivers remain limited. In this study, we used Soil Degradation Proxy (SDP), that integrates four key indicators of soil degradation, including erosion rate, soil pH, electrical conductivity, and organic carbon content, to quantify soil degradation risk. Using over 38,000 LUCAS topsoil observations and a machine learning model trained on climate, land cover, topographic, soil parent material properties, and spectral variables, we map annual SDP values between years 2000 to 2022 across Europe. Results show soil degradation risk is highest in southern Europe, especially in intensively managed and sparsely vegetated landscapes. Over the past two decades, approximately 7.1% of land area across the EU and the UK has experienced increasing degradation risk (most notably across Eastern Europe), with rainfed croplands emerging as the most affected land cover type. Land cover is the most influential driver, modulating effects of climatic variables such as precipitation and temperature on SDP. This data-driven framework provides a consistent and scalable approach for monitoring soil degradation risk and offers actionable insights to support targeted conservation and EU-wide policy implementation.

**Supplementary Information:**

The online version contains supplementary material available at https://doi.org/10.1038/s41598-025-33318-7.

## Introduction

Soil degradation refers to any change or disturbance to the soil that is perceived to be deleterious or undesirable^1^. Framing soils in terms of degradation simplifies assessment by reducing the problem to a binary choice, where the focus is on identifying whether soil threats are present or absent, and soils without any threats are then considered healthy^2^. Soil threats adversely affect soil health and impact ecosystem service delivery, such as nutrient cycling, the water balance, and carbon sequestration^3–5^. Erosion, compaction, loss of soil organic carbon, salinization, contamination, and loss of soil biodiversity are commonly recognized as major soil degradation threats^6^; these have been exacerbated by the recent changes in global land use patterns^7^.

It is necessary to assess, mitigate, and manage soil degradation at different spatio-temporal scales, as it threatens agricultural productivity, (sub-)surface biodiversity, and environmental sustainability^8^. The need for addressing soil degradation is also stressed by climate change projections, which suggest that we are moving toward a more vigorous hydrological cycle^9,10^ with plausible substantial increases in erosion rates at the global scale^11^.

Effective assessment methodologies are essential for understanding the extent of soil degradation and informing targeted interventions to mitigate its impacts^12^. Lehmann et al. (2020)^13^ opined that, ‘*creating a soil-​health index is difficult*,* as indicated by the relatively low number of published indices*,* because it requires quantitative transformation and weighting of multiple indicators*,* including categorical properties*,* to integrate them into a final single score.*’ In an analysis of their database, with more than 500 studies, only five attempted to provide an operational index. Part of the problem is the definition of soil quality as the ‘capacity to function’, which implies a fitness for use framing of quality. In this framing, operationalizing requires both the assessment of function and the criteria to evaluate soil performance. Function is rarely measured directly and relies on identifying appropriate indicators, while performance depends on intended ‘use’, which can narrow the quality assessment, usually to agricultural soils^14–16^. This has led recent work to explore alternative quality assessment frameworks, including ‘benchmarking’^17^ and what might be considered a modified quality control approach based on the frequency of degradation^18^.

Various studies have been conducted to explore soil degradation levels using different approaches and tools, including counting the number of specific soil threats occurring over a region^2,18–20^, assessing trends in soil degradation indicators aligned with sustainable development goals (SDG 15.3.1)^21,22^, and combining multiple soil degradation indicators by employing fuzzy logic-based techniques^23^. These studies have provided useful tools for informed decision-making and understanding soil threats at different spatial levels, from regional to continental scales. However, the subjective thresholds defined in their methodology introduce a binary assessment of degradation^18,24^, when it will be more nuanced, resulting in variability in identifying degraded areas, as the extent of soil threats can vary significantly based on the chosen thresholds. Additionally, the binary nature of the implemented approaches, with a simplistic categorization of soil threats to presence or absence, might fail to capture the complexity of soil degradation; this leads to a limited understanding of degradation intensity.

Harmonized surveys such as Land Use/Cover Area-frame Survey (LUCAS)^25^ provide continent-wide, protocol-consistent measurements of soil physicochemical properties, eliminating calibration bias and enabling direct comparison of conditions (e.g., soil pH level, salinity, SOC, nutrients) across national borders. However, these point observations remain sparse in space and time, leaving large knowledge gaps between sampling dates and locations. Increasing availability of remotely sensed observations and global climate reanalysis datasets enables machine-learning models to bridge these gaps by linking field measurements with spatially continuous environmental covariates. Moreover, explainable AI techniques applied to these models can clarify the local influence of individual predictors on degradation risk. The resulting outcomes, however, carry compounded uncertainties originating from sparse sampling, errors in gridded covariates, and model-related limitations that all propagate into the final products.

Despite extensive research on individual soil threats, Europe still lacks a harmonized, temporally resolved assessment that integrates multiple degradation processes in a single, continuous metric, utilizing the continent-wide consistency of LUCAS field data, and exploiting the utility of remotely sensed and climate information at fine spatial resolution. Current products, such as the map of the EUSO dashboard^2^ and Land Multi-degradation Index^18^, provide threshold-based soil degradation level snapshots, while most machine-learning studies focus on one threat at a time^11,26^. No study has yet combined the empirical strength of LUCAS with annual gridded covariates to deliver an annual sequence of continuous degradation risk estimates or the environmental controls behind them.

Aiming to provide a better understanding of soil-degradation patterns across Europe and a robust quantification of its evolution during the last two decades, this study first derives a continuous Soil Degradation Proxy (SDP) through integrating four indicators of soil degradation, including erosion rate, electrical conductivity, pH and soil organic carbon, representing different aspects of physical, chemical, and biological soil degradation processes. The SDP is then used as the response variable in a machine-learning framework that relates it to a set of climatic, spectral, topographic, lithological, and land-cover covariates. Model interpretation techniques are applied to quantify the marginal and context-specific influence of each predictor on SDP. The trained model is subsequently used to generate annual SDP maps for years 2000 to 2022, which are subjected to pixel-wise linear trend analysis to identify statistically significant areas of change in SDP over the past two decades.

## Results

### SDP distribution across Europe

**Fig. 1:**
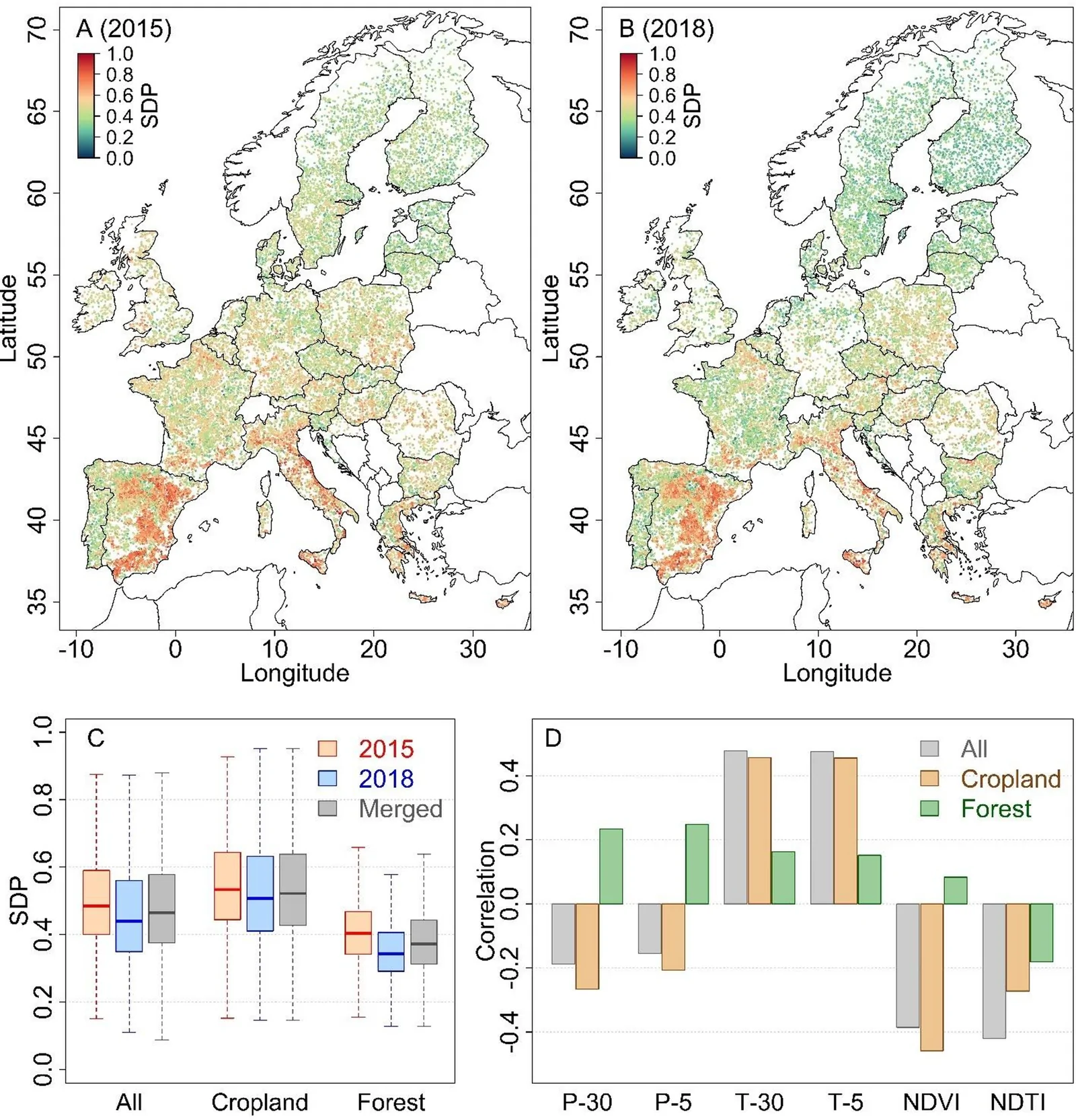
Spatial and statistical variations of SDP across LUCAS observations for the year 2015 and 2018. **A&B**, Spatial distribution of SDP across LUCAS observations for years 2015 and 2018, respectively. **C**, Variation of SDP across different land cover types. **D**, Correlation of SDP with different environmental variables. P-30: Long-term average of annual precipitation (last 30 years), P-5: Short-term average of annual precipitation (last 5 years), T-30: Long-term average of temperature (last 30 years), T-5: Short-term average of temperature (last 5 years), NDVI: Average NDVI between months May to August, NDTI: Average NDTI between months March to April.

**Fig. 2:**
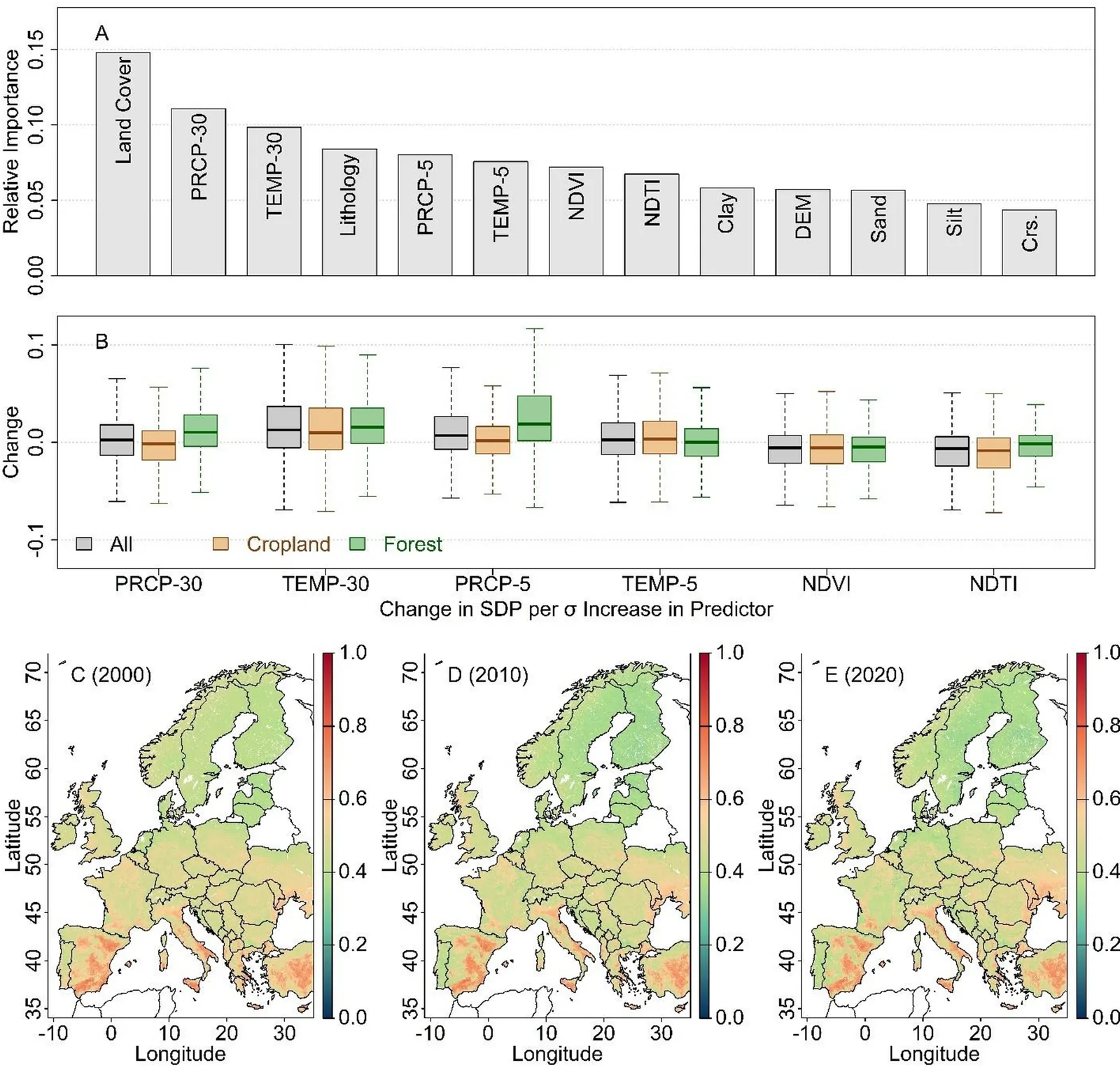
Evaluation of SDP predictions and environmental predictors of it using RF across Europe. **A**, Relative importance of environmental variables in prediction of SDP, **B**, Change in SDP per one standard deviation increase in environmental predictors of SDP, **C**, Predicted SDP map of the year 2000, **D**, Predicted SDP map of the year 2010, **E**, Predicted SDP map of the year 2020, PRCP-30: Long-term average of annual precipitation (last 30 years), PRCP-5: Short-term average of annual precipitation (last 5 years), TEMP-30: Long-term average of temperature (last 30 years), TEMP-5: Short-term average of temperature (last 5 years), NDVI: Average NDVI between months May to August, NDTI: Average NDTI between months March to April, Clay: Clay fraction of soil, Sand: Sand fraction of soil, Silt: Silt fraction of soil, Crs: Coarse fraction of soil DEM: Digital elevation model.

The spatial distribution of the SDP across Europe was calculated using LUCAS 2015 and 2018 topsoil data observations (Figs. 1A and 2B, with individual input indicators provided in Supplementary Fig. 1). The continent-wide analysis of SDP values based on LUCAS observations reveals a clear latitudinal gradient with higher SDP values observed in the Mediterranean regions. Countries such as Malta, Cyprus, Spain, and Italy display average SDP values ranging from 0.59 to 0.76. Moving northward, average SDP values progressively decline, with Estonia, Latvia, Finland, and Lithuania exhibiting the lowest levels between 0.35 and 0.39. These spatial patterns are also evident when the data are analyzed by land cover type, where forested areas show notably lower median SDP value of 0.37 compared to 0.52 in arable fields, supporting the role of trees in mitigating degradation (Fig. 1C).

The correlation analysis between different environmental factors and the SDP (Fig. 1D) shows that vegetation-based indices such as NDVI and NDTI exhibit contrasting effects depending on land cover type. While NDVI and NDTI show strong negative correlations with SDP in croplands (-0.46 and -0.27, respectively), they are weakly or even positively correlated in forests (0.08 and -0.18, respectively). Climatic drivers exert contrasting influences as well. Long-term precipitation (P-30) shows a negative correlation with SDP in croplands and a positive one in forests, while long‑term temperature exerts a uniformly positive effect across both covers.

### Model interpretability and drivers of SDP

The random forest (RF) models used for SDP prediction achieved, on average, an R-squared of 0.602 and mean absolute error (MAE) of 0.072 in the validation datasets. Expanded accuracy assessments are provided in the supplementary materials, including R-squared, root mean squared error (RMSE), and MAE for both training and validation datasets across the 100 bootstrap models (Supplementary Table 1), a predicted-versus-observed scatter plot for the validation dataset (Supplementary Fig. 2A), and boxplots summarizing prediction errors across ten SDP intervals from 0 to 1 (Supplementary Fig. 2B). However, it exhibited limitations in accurately predicting low SDP values particularly due to data scarcity in that range (less than 2.5% of observations are in the range of less than 0.25 SDP). The relative importance of various predictors (Fig. 2A) and sensitivity analysis of the model (Fig. 2B) reveal that land cover type, precipitation, temperature, and lithology have the highest influences on the modelled SDP. The response of SDP to a one standard deviation change (increase) in each predictor variable indicates that climate variables, both precipitation and temperature, have land cover-dependent impacts on SDP. An increase of one standard deviation in long-term precipitation decreased SDP in croplands but increased it in forests. A similar increase in temperature led to higher SDP values across all land cover types.

**Fig. 3:**
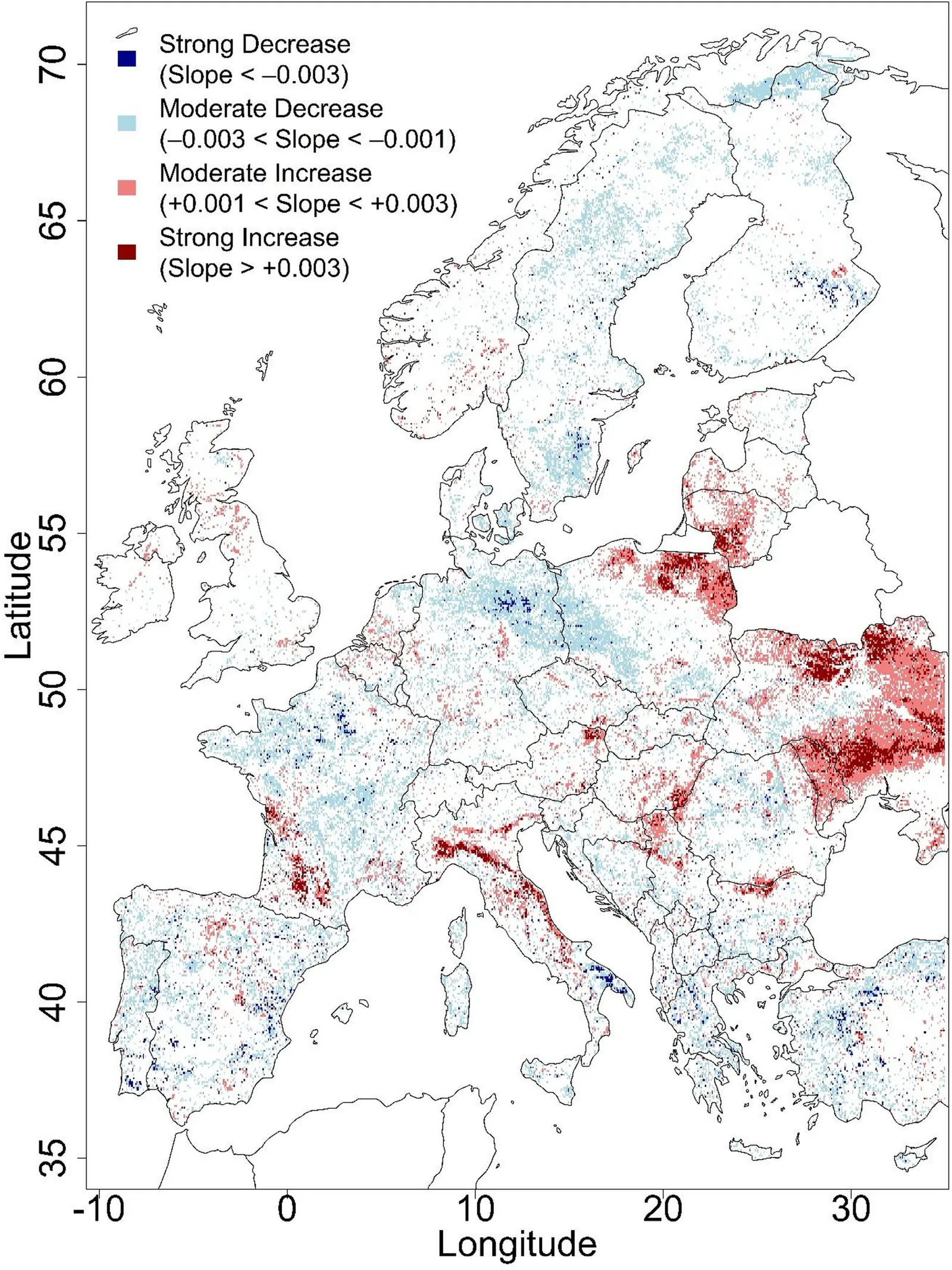
Trends of SDP across Europe between 2000 and 2022. The non-significant slopes (*p* > 0.01) are not shown. Positive slope shows an increase in SDP (going toward higher degradation risk and unhealthier soil), and negative slope shows a decrease in SDP (going toward lower degradation risk and healthier soil).

Applying this model retrospectively yields continent‑wide SDP maps between years 2000 to 2022 that capture spatial heterogeneity consistent with the 2015–2018 surveys (Fig. 2C and E; corresponding uncertainty maps derived from the 100-member bootstrap ensemble are provided in Supplementary Fig. 3). Trend analysis over the 2000–2022 SDP stack indicates that 7.2% of Europe’s land surface has experienced a moderate rise in SDP (slope > 0.001), with increases concentrated in rainfed croplands (17.1% of rainfed croplands show a moderate and 4.1% a strong upward trend; slope > 0.003). Forests are comparatively stable, with only 1.6% and 0.05% of their extent experiencing moderate and strong increases, respectively (Fig. 3).

**Fig. 4:**
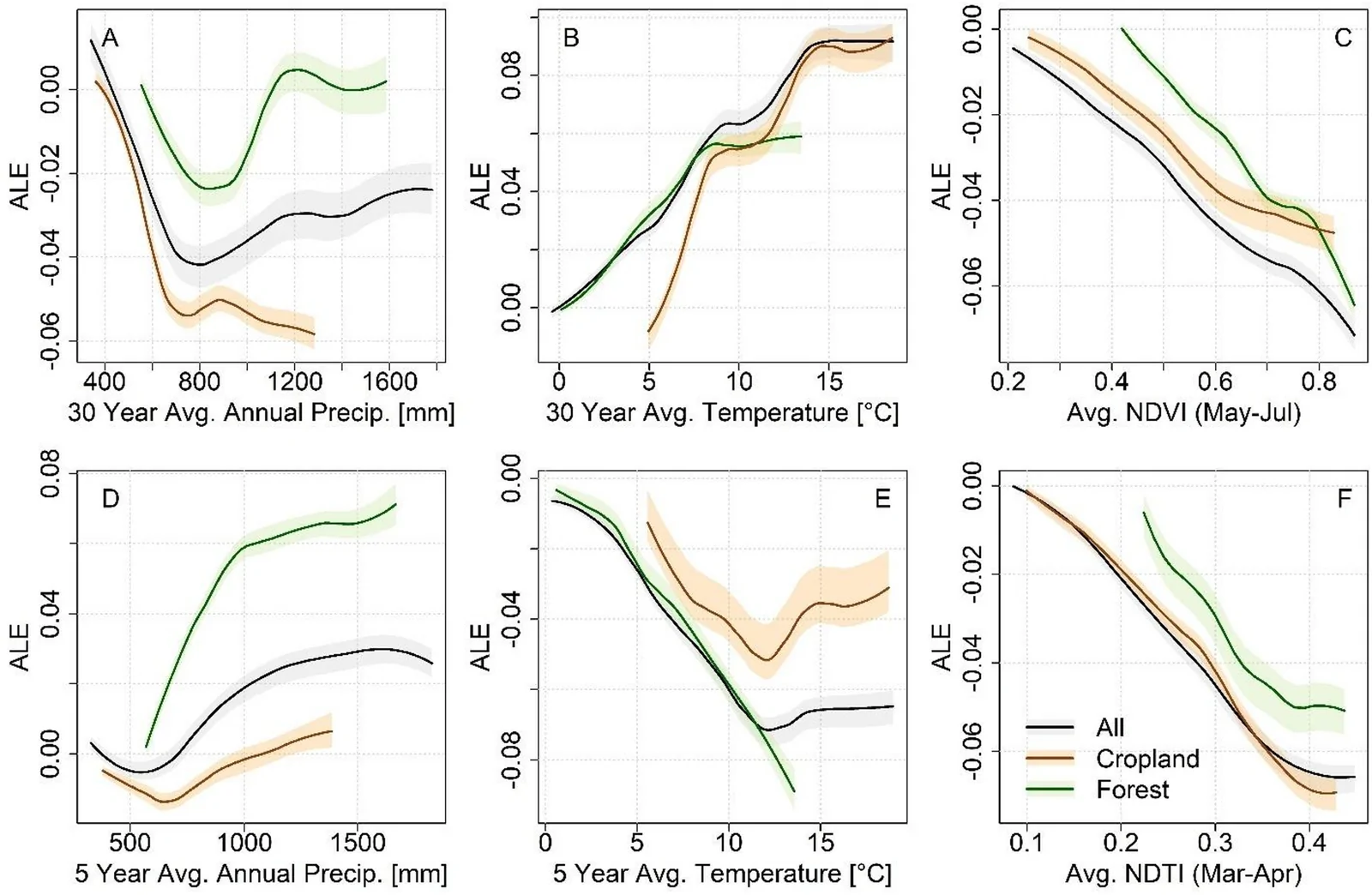
Accumulated Local Effects (ALE) of various environmental variables on the SDP for cropland (brown) and forest (green) areas, with average predictions of 0.48, 0.53, and 0.38 over all landcovers (All), cropland, and forest areas, respectively. The uncertainty bounds are obtained by running the same analysis over 50 bootstrap samples. Avg: Average, Precip: Precipitation.

The multifaceted interactions between climatic variables, and land management practices in determining soil degradation risks are further explained by the accumulated local effects (ALE) of various environmental factors on the SDP (Fig. 4). For long-term precipitation (Fig. 4A), forests exhibit a U-shaped pattern, where SDP initially decreases with increasing precipitation but begins to rise again beyond ~ 800 mm/year. A similar non-linear behavior could be observed for short-term precipitation (Fig. 4D). SDP in croplands decreases under moderate rainfall levels but increases beyond ~ 700 mm, indicating that excessive rainfall events can elevate degradation risk through enhanced runoff and erosion. Forested areas, meanwhile, display a steady rise in SDP with increasing short-term precipitation, reflecting their susceptibility to slope instability under very wet conditions. In contrast, croplands show a consistent direct relationship between precipitation and SDP. The effects of temperature also vary depending on land cover type and temporal scale (Fig. 4B and E). Croplands are more responsive to short-term average temperatures, with SDP values initially decreasing under moderate warming up to ~ 12 °C likely due to enhanced microbial activity and increased biomass production. However, beyond this threshold, degradation risks increase, perhaps driven by heat stresses on crops, reduced moisture retention, and accelerated organic matter decomposition^27,28^. Vegetation-related indices such as NDVI (Fig. 4C) and NDTI (Fig. 4F) show strong and consistent negative associations with SDP, particularly over croplands.

## Discussion

The spatial patterns derived from observed SDP values based on LUCAS topsoil data reveal a clear north-to-south contrast across Europe (Fig. 1A and B). This gradient corresponds well with established climatic, land cover, and soil characteristics that influence soil vulnerability to degradation across Europe. Higher SDP values in southern Europe are likely driven by arid to semi-arid conditions, frequent erosive rainfall, and a long history of intensive cultivation on inherently carbon-poor soils^11,29^. In contrast, lower SDP values in northern and central Europe may result from more temperate and humid climates, denser vegetation cover, and generally greater soil protection under forest and grassland systems^30^.

Compared to previous European-scale assessments of soil degradation^2,18^, our approach offers a more flexible and data-driven framework. While the spatial patterns of SDP values broadly align with the concurrent degradation processes of Land Multi-Degradation Index (LMI)^18^, the methodology used to develop the LMI relies on binary classification of soils as either healthy or degraded based on predefined thresholds, which can introduce subjectivity and limit temporal scalability of it. In contrast, SDP provides a less subjective and continuous representation of degradation risk, capturing gradual transitions between healthy and degraded soils. Moreover, our framework links the harmonized LUCAS soil dataset with annually varying climatic, spectral, and land-cover covariates. This integration enables the temporal tracking of soil degradation risks across Europe. As a result, it provides a more consistent and scalable monitoring system, particularly useful for policy applications aligned with the EU Soil Deal.

Land cover plays a critical role in modulating these climatic pressures. Forests, with their dense canopy structures and deeper rooting systems, enhance soil cohesion and reduce exposure to erosive forces^31^. In contrast, arable lands leave soils more vulnerable by exposing surfaces to rainfall impact, tillage disturbance, and seasonal vegetation gaps (Fig. 1C). Model results further support these dynamics. In croplands, lower SDP values (indicating reduced degradation risk) were consistently associated with higher NDVI (representing active vegetation cover; Fig. 4C) and NDTI (representing crop residues; Fig. 4F) values, reflecting the importance of management practices and residue retention in mitigating soil degradation^32^.

The model also reveals that the effects of precipitation and temperature on SDP are land cover dependent. In croplands, increased precipitation generally improves soil health by supporting biomass growth and moisture retention. However, in forested areas, excessive rainfall can exacerbate runoff, leaching, and slope destabilization (Fig. 4A). While forests typically offer greater soil protection under moderate rainfall conditions, the model captures a localized increase in degradation risk under extreme precipitation^33^. The positive relationship between long-term temperature and SDP across land covers (Fig. 4B) suggests that warming climates may negatively impact soil moisture, organic matter dynamics, and overall soil health^34^. However, the response of SDP to short-term (5-year) temperature variability shows a different pattern. SDP values decrease up to approximately 12 °C, suggesting that moderate warming may enhance plant productivity and microbial activity, leading to temporarily improved soil conditions (Fig. 4E). Beyond this range, however, SDP begins to rise again, indicating that further temperature increases likely induce heat stress, accelerate organic matter decomposition, and reduce soil moisture retention, implying the non-linear effects of short-term warming on soil degradation^27,35^.

Trend analysis from 2000 to 2022 indicates increasing degradation risk in agricultural systems, particularly in rainfed croplands (Fig. 3). This temporal pattern aligns with regional case studies across Europe^36^. In southwestern France, long-term monitoring has documented organic matter depletion and erosion in agricultural soils^36^. Across the Baltic states, unsustainable practices have intensified degradation processes such as compaction and contamination^37^. The model’s ability to capture these broad yet regionally consistent trends demonstrates the value of combining machine learning with environmental predictors to reveal both spatial and temporal degradation dynamics.

While the developed model effectively captures broad spatial and temporal patterns of SDP across Europe, some limitations remain. The model showed slightly reduced accuracy in areas with very low SDP values, particularly under dense forest canopies. This may reflect the limited sensitivity of certain remotely sensed indices in such settings, as well as the smaller number of observed low-degradation sites in the dataset. Given the continental scale of analysis, spatial autocorrelation among environmental predictors (e.g., precipitation, temperature, vegetation indices) may also cause some relationships to appear stronger than they actually are. The influence of environmental predictors on soil degradation can vary regionally, depending on local soil and landscape characteristics that are not fully captured at continental resolution. In addition, part of the predictive uncertainty stems from variability introduced during model training, including differences in predictions associated with the sampling of training data (Supplementary Fig. 3). Some of the remaining model errors may also originate from inaccuracies in the gridded input variables employed as predictors. Addressing these challenges may require the inclusion of indicators better suited to capturing subtle variations in soil and vegetation conditions (e.g., Sentinel-1 VV/VH backscatter or Sentinel-2 red-edge-based indices such as normalized difference red edge; NDRE), along with more extensive and targeted data collection campaigns in ecosystems where degradation is less visually apparent. Future refinements could also benefit from stratifying the analysis into more homogeneous environmental zones to reduce spatial dependence and improve interpretability.

The development of the SDP from multiple SHIs also warrants consideration as a methodological choice. In this study, we selected SHIs that capture complementary aspects of soil degradation. Equal weights were applied to all SHIs to ensure continent-wide comparability and to avoid introducing subjective biases in the absence of universally agreed weighting criteria. Nevertheless, the relative importance of different SHIs is likely to vary with landscape context, soil type, or climate. Considering different SHIs and assigning weights to them, potentially guided by expert knowledge or data-driven approaches, could allow the SDP to more accurately reflect local degradation risks. Future research may explore how such weighting schemes can be adapted on landscape context or soil district^38^ characteristics, thereby enhancing the SDP’s relevance while maintaining scientific comparability.

Further work could also explore the integration of soil district delineations into predictive modeling frameworks. The ALE plots in this study indicate that the response of SDP to environmental drivers varies considerably across different pedo-ecological contexts. Grouping areas with similar soil, climate, and landscape conditions, could be helpful in reducing the influence of spatial autocorrelation and non-stationarity that affect continental-scale models, allowing predictor relationships to be calibrated locally and interpreted more reliably. Soil districts, defined by shared characteristics such as soil type, topography, and land use, may offer operational spatial units that improve model accuracy and facilitate the translation of results into local-scale management strategies.

The findings from this study support the use of data-driven tools to inform soil conservation strategies across Europe. Within the areas covered by the LUCAS observations, the model reliably identifies regions with increasing degradation risk, offering a robust evidence base for proactive management interventions and policy prioritization. The spatially explicit predictions and long-term trends produced by the model can aid environmental reporting, guide land-use planning, and support the implementation of the EU Soil Monitoring and Resilience Directive. Connecting these scientific outputs with governance structures, including administrative boundaries, will be essential for translating risk assessments into both local action and EU-wide policy responses.

## Concluding remarks

This study introduces a new data-driven framework for assessing soil degradation risk across Europe through the development and application of the SDP. The SDP was developed using four indicators of soil degradation, including erosion rate, soil pH, EC, and SOC to provide a continuous and spatially consistent measure of soil vulnerability to degradation. Leveraging a machine learning model trained to link SDP to different environmental and land cover predictors, we generated spatial predictions of SDP across Europe and evaluated its long-term dynamics between 2000 and 2022.

Beyond its methodological contribution, the developed approach offers valuable implications for soil policy, management practices, and environmental reporting. The SDP provides a harmonized basis for identifying degradation hotspots, tracking progresses toward soil degradation neutrality targets, and supporting implementation of the EU Soil Monitoring and Resilience Directive and the Soil Deal for Europe. Its capacity to integrate diverse environmental predictors also facilitates early warning systems for soil degradation.

Looking forward, further work could focus on coupling the SDP with local soil districts delineations to refine regional assessments, incorporating additional indicators of biological and structural soil health, and expanding the framework to predict future degradation trajectories under policy or management scenarios. Linking the developed framework in this study with actionable policy instruments can contribute in building a coherent European soil information system that would support sustainable land management and long-term soil resilience.

## Methods

### Soil degradation proxy

Soil quality, defined as ‘capacity to function’, conceptualizes the quality framework on ‘fitness for purpose’^39^. Challenges in operationalizing this are the need to define, *who the purpose is for*, and what constitutes *fitness* for purpose. A major challenge in the case of soil sustainability is that what is ‘good’ under one land use might be ‘bad’ under another. Hence, this approach is usually constrained by focusing on a specific land use. This ‘fitness for purpose’ approach is embodied in the well-established Cornell Comprehensive Assessment of Soil Health, which frames quality in a largely agricultural context. It goes on to define fitness by assigning values between 0 and 100 using indicators which are interpreted essentially using three categories of ‘More is better’, ‘Optimum curve’, and ‘Less is better’^40^.

Conversely, the degradation approach taken in this work is more consistent with the ‘zero defects’ concept, i.e. a soil without degradation is viewed as the minimum acceptable state to be achieved^39,41^. This approach underpins traditional quality control, and thus, quality control approaches and tools are applicable^43^. We adopt the general categorization for assessing degradation threats of, ‘More is better’, ‘Optimum curve’ and ‘Less is better’^40^ to develop a new SDP which provides both space and magnitude of the degradation, identify spatial locations to improve and determine the key drivers, communicate to land managers and decision makers to plan interventions to mitigate the degradation.

SDP combines four indicators of soil degradation, including erosion rate, electrical conductivity (EC), soil organic carbon (SOC), and pH, which have been used often in globally assessed soil degradation studies^18,20^. These variables form the smallest set that covers the three major degradation domains of physical (erosion), chemical (salinity and acidity/alkalinity), and bio-chemical (SOC and acidity/alkalinity). Moreover, each of these properties is obtained from sources that are consistent among sampling points, providing continent-wide consistency. Furthermore, erosion rate, EC, pH, and SOC can all be related to climate and remotely sensed covariates with proven skill, allowing us to reconstruct their behavior for years when field data are absent. Soil erosion reflects the loss of topsoil and highlights the disruption of soil structure and stability as a result of natural and anthropogenic activities^42^. High erosion rates adversely affect agricultural productivity and the ability of soil to perform its ecological functions^43–45^. Soil salinity can negatively impact plant water uptake^46^, reduce soil fertility^47^, and cause toxic accumulations in soil that constrain plant growth^48^. Soil EC is commonly used to determine the level of soluble salts in the soil^26^. In salt-affected landscapes, salinity has been reported to cut the average yield of major crops by more than 50%^49^. Soil pH, a bio-chemical indicator of soil health, has a profound impact on nutrient availability and microbial activity^50–52^. Both high and low soil pH levels result in nutrient deficiencies, reduced microbial activity, decreased crop yields, and overall deterioration of soil health^53^. While our model employs a single pH optimum for practical purposes, we acknowledge that optimal pH ranges vary considerably across different ecosystems and agricultural systems. Moreover, SOC provides comprehensive information about soil degradation across physical, chemical, and biological properties^54^. Its presence contributes to improved soil structure^55^, water retention^56^, nutrient cycling^57^, fertility^58^, and overall soil health^59,60^. Similarly, while SOC generally indicates soil quality, optimal levels are ecosystem-dependent, and high-SOC environments such as peatlands can remain vulnerable to erosion and degradation. Other potential indicators (e.g., bulk density, soil contamination), were considered but excluded because they are not measured systematically in LUCAS, which would compromise the temporal-trend analysis conducted in this study.

These indicators provide valuable insights into the dynamic nature of soil health, offering diverse perspectives on erosion risk, salinity, nutrient content, and soil acidity and alkalinity levels. By integrating these indicators in a continuous way, the SDP offers a refined understanding of degradation intensity across different landscapes and environmental conditions.

Within the SDP calculations, the four soil health indicators are required to be rescaled to a range of 0 to 1. This rescaling process involves mapping of observations to values between zero to one based on their cumulative distribution function (CDF) values, with assigning a value of zero to the minimum and one to the maximum value of the observed values (Supplementary Fig. 1). Notably, this relationship establishes a positive relationship between SDP and both soil erosion rate and EC, emphasizing their enhancing role in soil degradation. Conversely, an inverse relationship is defined between SDP and SOC, reflecting the protective influence of higher SOC levels against soil degradation. Furthermore, pH values are bidirectionally linked with the SDP, with a pH of 6.5 serving as the pivotal point^61–63^. Following this bidirectional relationship, the SDP worsens as pH values diverge towards both ends of its scale. Equation one illustrates this relationship:

1 $$\:{SDP}_{i}=\frac{{SHI}_{i}^{ER}+{SHI}_{i}^{EC}+{SHI}_{i}^{pH}+{SHI}_{i}^{SOC}}{4}$$

Where, SDP_i_ is the soil degradation proxy index of sample “i” among considered observations, SHI^ER^ and SHI^EC^ are soil health indicators (SHI) obtained from rescaled values of erosion rate and electrical conductivity of sample “i” (CDF value of erosion rate and electrical conductivity of sample “i”; ‘more is worse’), SHI^pH^ is the SHI obtained from rescaled form of pH value of sample “i” (CDF value of pH of sample for pH values greater than 6.5 and one minus CDF value of pH of sample for pH values less than 6.5; ‘inverse optimum’, where ~ 6.5 is a mid-point between pH 4.9 and 8.1^64^, and SHI^SOC^ is the SHI obtained from rescaled form of soil organic carbon value of sample “i” (one minus CDF value of soil organic carbon of sample “i”; constructed so that lower SOC results in higher SHI values, i.e., ‘more is worse’). The factor 1/4 in Eq. 1 ensures that the final SDP value remains between 0 and 1, with each SHI contributing equally to the SDP with a weight of 0.25^2,18,20^.

### Datasets

**Fig. 5:**
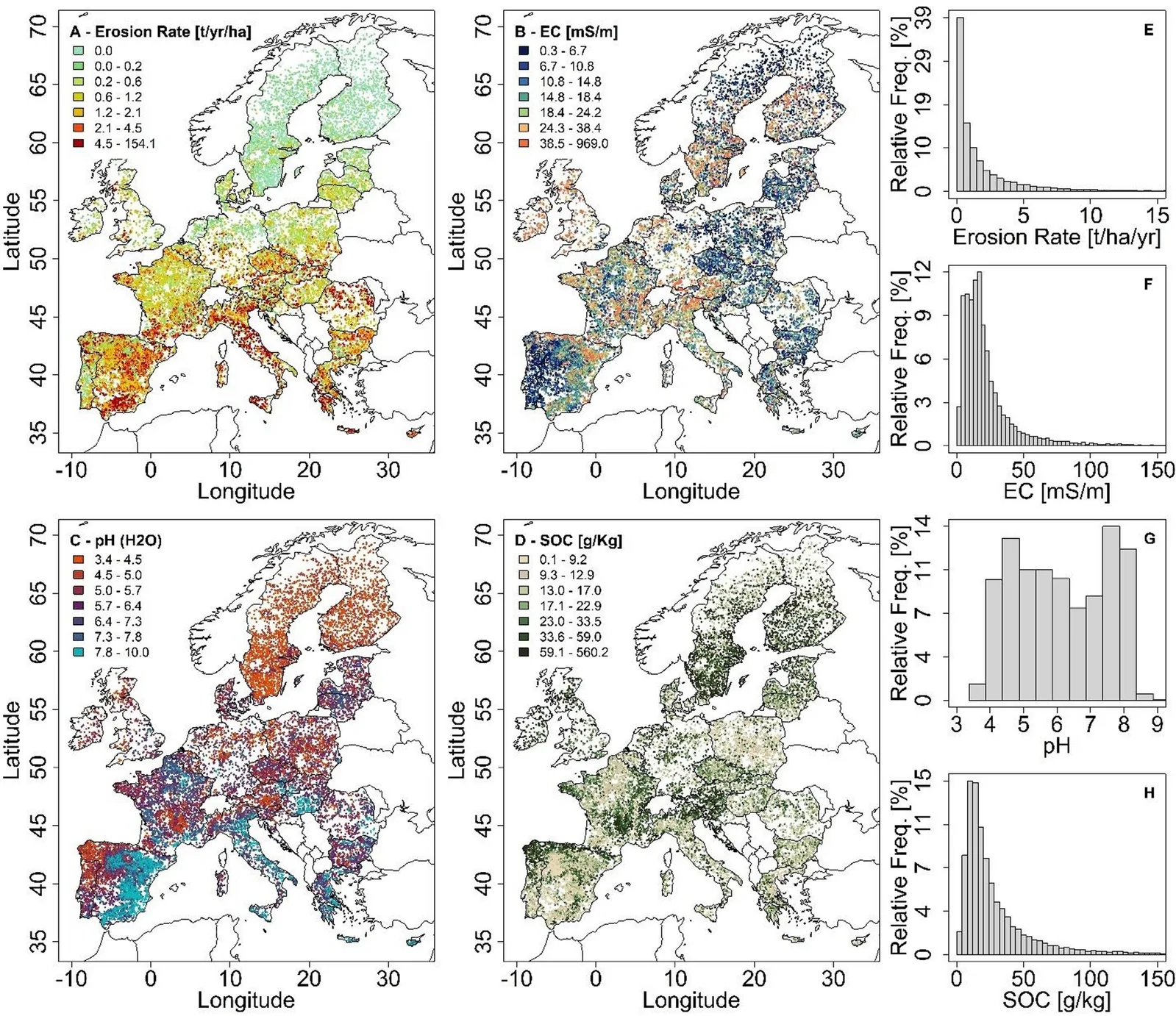
Spatial distribution of soil erosion rate (**A**), electrical conductivity (EC; **B**), pH (**C**), and soil organic carbon (SOC; **D**) over LUCAS observations. Panels **E-H** show the relative frequencies of erosion rate for the year 2015, EC, pH, and SOC, respectively, as a percentage of the total observations. The data in panels A–D are classified using quantile-based intervals. The upper limit of the X-axis value in panels E-H is set at the 99th percentile for each variable.

We used LUCAS datasets^25^, a periodic EU-wide monitoring program, to develop CDFs of the four components considered in the calculation of SDP across Europe. While the soil erosion data were extracted from the Revised Universal Soil Loss Equation (RUSLE) model (RUSLE2015)^65–70^, the other three components of SDP including soil EC, soil SOC, and soil pH are obtained from LUCAS observation points of the year 2015 and 2018. Due to the unavailability of soil erosion data for the years 2015 and 2018, we assumed erosion rates remained constant and used the same extracted values from the RUSLE2015 model for both years. The spatial distribution of four components considered in the development of SDP and their relative frequencies over the LUCAS observations of the year 2015 are shown in Fig. 5.

We also used a comprehensive set of predictors related to environmental and soil-related factors, sourced from multiple data repositories to capture different aspects of soil degradation within the RF model development framework (Table 1). The input variables included Digital Elevation Model (DEM)^71^, land cover types (sourced from Copernicus datasets)^72^, and lithology^73^ maps. Climate variables, including precipitation and temperature, which are obtained from ERA5-Land datasets^74^, provide annual sum and average values over the last 30 and 5 years prior to the observation year. Additionally, average Normalized Difference Vegetation Index (NDVI) between months May and August (for representing vegetation density and health)^75^, as well as average Normalized Difference Tillage Index (NDTI)^76^ between months March and April (for representing crop residues) were calculated from MODIS satellite imagery by averaging the respective monthly products over each period. NDTI provides information on the proportion of non-photosynthetic vegetation and exposed soil, thereby complementing NDVI in capturing surface conditions related to land management and soil disturbance^77^. Soil texture percentages, comprising coarse, clay, sand, and silt fractions, were obtained from soil grid datasets^78^. We acknowledge that some inconsistencies may exist between gridded soil datasets and in-situ observations. However, their accuracy for inherent soil properties (e.g., sand, silt, and clay fractions) has shown to be generally acceptable for large-scale modeling applications^79^. Hence, given the continental scope of this study, gridded datasets were employed to ensure complete and consistent coverage across the study area.

All above-mentioned variables are sampled over LUCAS sampling points of the years 2015 and 2018 to be utilized in the training of the RF model. Upon completion of the training process, raster datasets from various sources were resampled to a spatial resolution of 1 km to ensure consistency and facilitate the application of the RF model to generate annual SDP maps across Europe. The spatial extent of the analysis was defined by the overlap of all environmental predictor datasets to ensure consistent data coverage, spanning approximately 11° W to 35° E longitude and 34° N to 72° N latitude. Regions beyond these limits were excluded due to either boundary constraints or incomplete predictor data.

**Table 1:** The variables considered as predictors of SDP along with their corresponding abbreviations, sources, and Spatial resolutions.

Input Variable	Description	Source	Resolution
DEM	Average DEM within 100 m buffer	Copernicus [71]	100 m
PRCP	Average precipitation over the last 30 and 5 years	ERA5 Land [74]	~ 10 km
TEMP	Average temperature over the last 30 and 5 years	ERA5 Land [74]	~ 10 km
NDVI	Average NDVI between May to August	MODIS [75]	500 m
NDTI	Average NDTI between March to April	MODIS [76]	500 m
Coarse	Coarse fragments fraction of soil (> 2 mm; topsoil)	Soil Grids [78]	250 m
Clay	Clay fraction of soil (topsoil)	Soil Grids [78]	250 m
Sand	Sand fraction of soil (topsoil)	Soil Grids [78]	250 m
Silt	Silt fraction of soil (topsoil)	Soil Grids [78]	250 m
Lithology	Surface lithology class	EGDI / OneGeology [73]	250 m
Landcover	Land cover class	Copernicus [72]	100 m

### Exploratory statistical analyses

The variation of SDP across different land cover types (i.e., cropland, forest, and all land covers combined) was examined to describe how land cover types influence soil degradation risk patterns. Moreover, the correlations between SDP and selected environmental predictors, including vegetation indices and climatic factors, were analyzed to identify general patterns linking soil degradation risks with vegetation activity and climate conditions (shown in Fig. 1; panels C and D).

### Predictive modelling of SDP

An RF model was employed to establish the link between the SDP and soil degradation predictors. Combining available LUCAS observations over the 2015 and 2018 datasets built up 38,724 observations in total (19996 and 18728 for the years 2015 and 2018, respectively). This dataset was then split into a 70% and 30% ratio for training and validation portions. Moreover, to quantify predictive uncertainty associated with the RF model, we implemented a bootstrap-based ensemble approach. One hundred bootstrap replicates of the training dataset (sampling with replacement from the combined 2015–2018 LUCAS observations) were generated, and for each replicate an independent RF model was trained using the same predictor set. Each model was then applied to generate annual SDP predictions. For every pixel, the 0.05 and 0.95 percentile values of the 100 ensemble predictions were computed, and their difference was used as an estimate of model uncertainty.

The evaluation of the accuracy of the RF model in reproducing SDP observations across Europe has been done by comparing the predictions of the RF model with observed SDP values using the coefficient of determination (R-Squared), RMSE, and MAE. For each of the 100 bootstrap RF models, we calculated R-Squared, RMSE, and MAE for both the training and validation subsets. Moreover, to examine how model performance varies across the degradation-risk spectrum, SDP values in the validation dataset were grouped into ten equal intervals, and the distribution of prediction errors within each bin was summarized using boxplots. Furthermore, a predicted-versus-observed scatter plot was generated to illustrate model behavior and residual structure.

Different soil degradation predictors, representing topography, climate, soil parent material properties, along with remotely sensed lithology (gap-filled to ensure complete continental coverage), and land cover type properties, were used as inputs in the developed model. Variable importance was computed by quantifying the contribution of each predictor in reducing the overall model error using the mean decrease in node impurity (Gini importance) metric. To further quantify the sensitivity of SDP to individual predictors, we evaluated the model response to a one-standard deviation increases in each variable. For each predictor, all other variables were held constant while the focal variable was perturbed by one standard deviation. The resulting change in predicted SDP was recorded as an estimate of marginal effect magnitude (shown in Fig. 2B). Following this, the ALE analysis^80^ was performed to estimate the average effect of predictors by integrating their local influence on model predictions across the range of observed values. The ALE analysis was preferred over partial dependence plots to avoid bias from correlated predictors. Moreover, to quantify uncertainty, the ALE computation was repeated over 50 bootstrap resamples of the training data (with replacement in sampling). Finally, for each bootstrap, ALE curves were recalculated, and 95% confidence intervals were obtained from the distribution of bootstrap estimates. It is noteworthy that all above-mentioned analyses were performed by using Google Earth Engine^81^ and different R packages^82–87^ in the R environment^88^.

**Fig. 6:**
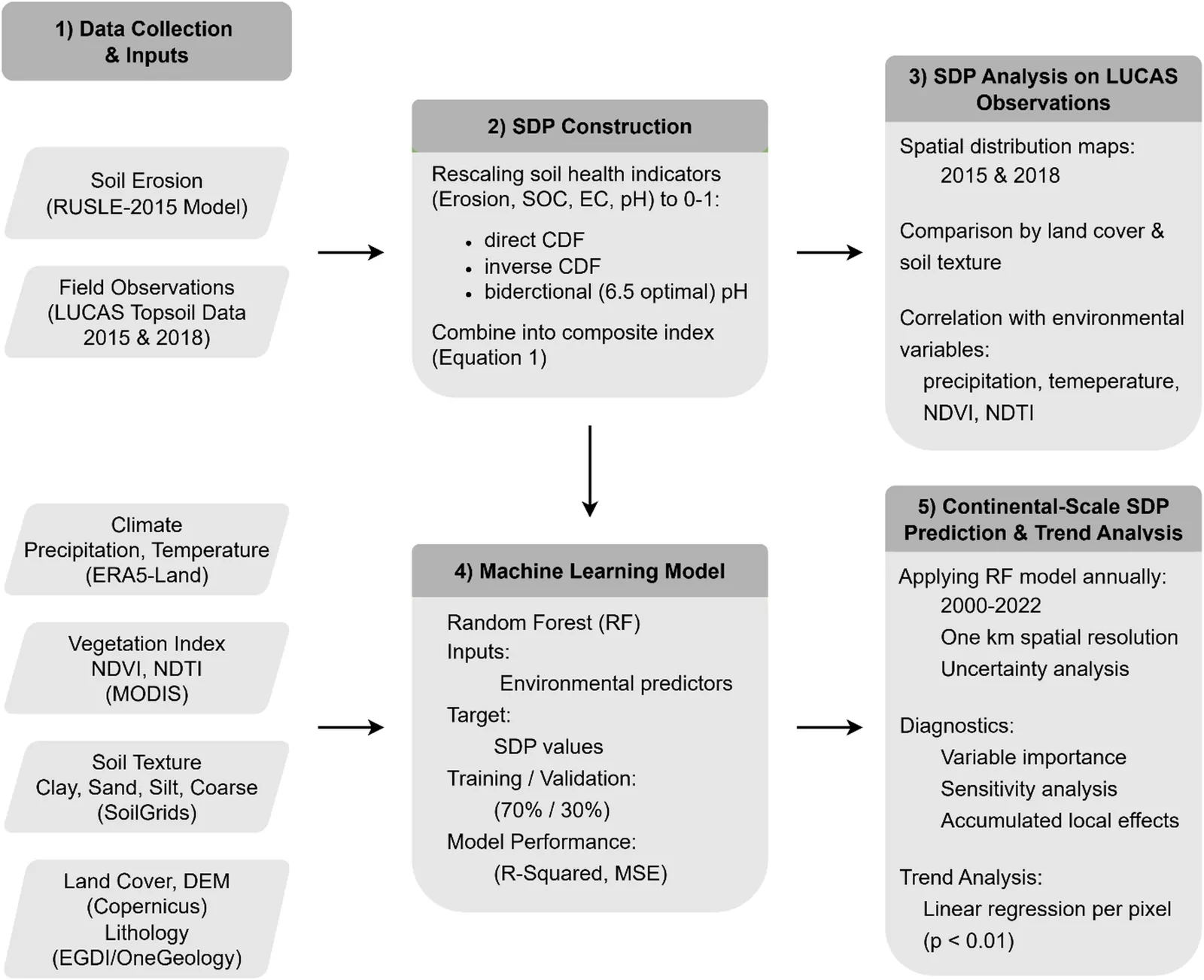
Overview of the methodological workflow used in this study for assessing SDP across Europe. LUCAS: Land Use/Cover Area frame Statistical Survey, RUSLE: Revised Universal Soil Loss Equation, SDP: Soil Degradation Proxy, SOC: Soil Organic Carbon, EC: Electrical Conductivity, CDF: Cumulative Distribution Function, RF: Random Forest, DEM: Digital Elevation Model, NDVI: Normalized Difference Vegetation Index, NDTI: Normalized Difference Tillage Index, R-Squared: Coefficient of Determination, MSE: Mean Squared Error.

Moreover, to examine long-term dynamics of SDP, we applied trend analysis to the annual SDP maps generated by the trained RF model between the years 2000 and 2022. The RF model was trained using LUCAS 2015 and 2018 topsoil observations, for which SOC, pH, and EC observations were available, and applied to annual environmental predictor layers (Table 1) covering the period 2000 to 2022 to estimate temporal changes of SDP. This analysis was conducted at the pixel level by computing the linear slope of SDP values over time, using ordinary least squares (OLS) regression with considering a significant level of *p* < 0.01 for the obtained slopes. The p-values were derived from the OLS regression t-statistics for the slope coefficients. To facilitate interpretation, we categorized significant slopes into “moderate” and “strong” trends based on magnitude thresholds. Moderate increase or decrease corresponded to absolute slope values between 0.001 and 0.003 SDP units per year, while strong trends exceeded 0.003 SDP units annually. Non-significant slopes (*p* ≥ 0.01) were masked out in the final trend maps. An overview of the data sources, processing steps, modeling approach, and analytical components of this study, is provided in Fig. 6.

## Supplementary Information

Below is the link to the electronic supplementary material.

Supplementary Material 1

Supplementary Material 2

## Data Availability

The required data of the study explained in Sect. Results were obtained from the following sources: The LUCAS observations are available at https://esdac.jrc.ec.europa.eu/content/lucas2015-topsoil-data, elevation data are obtained from https:/doi.org/10.5270/ESA-c5d3d65, MODIS observations are retrieved from https://doi.org/10.5067/MODIS/MOD11A2.061; https://doi.org/10.5067/MODIS/MOD13A2.061; and https://doi.org/10.5067/MODIS/MOD09GA.061, soil parent material properties were downloaded from SoilGrids https://doi.org/10.17027/isric-soilgrids.713396fa-1687-11ea-a7c0-a0481ca9e724, land cover datasets are taken from Copernicus Global Land Service https://doi.org/10.24381/cds.006f2c9a, and lithology maps are obtained from https://doi.org/10.5281/zenodo.12607973.
