# Supplementary material for: Spatial and temporal assessment of soil degradation risk in Europe
Source: Sci Rep. 2025 Dec 24;15:44636. doi: 10.1038/s41598-025-33318-7 (PMC12749955; doi:10.1038/s41598-025-33318-7)
Supplement: Supplementary file 1 — Supplementary Material 1 [file 41598_2025_33318_MOESM1_ESM.docx]

**Supplementary Materials**


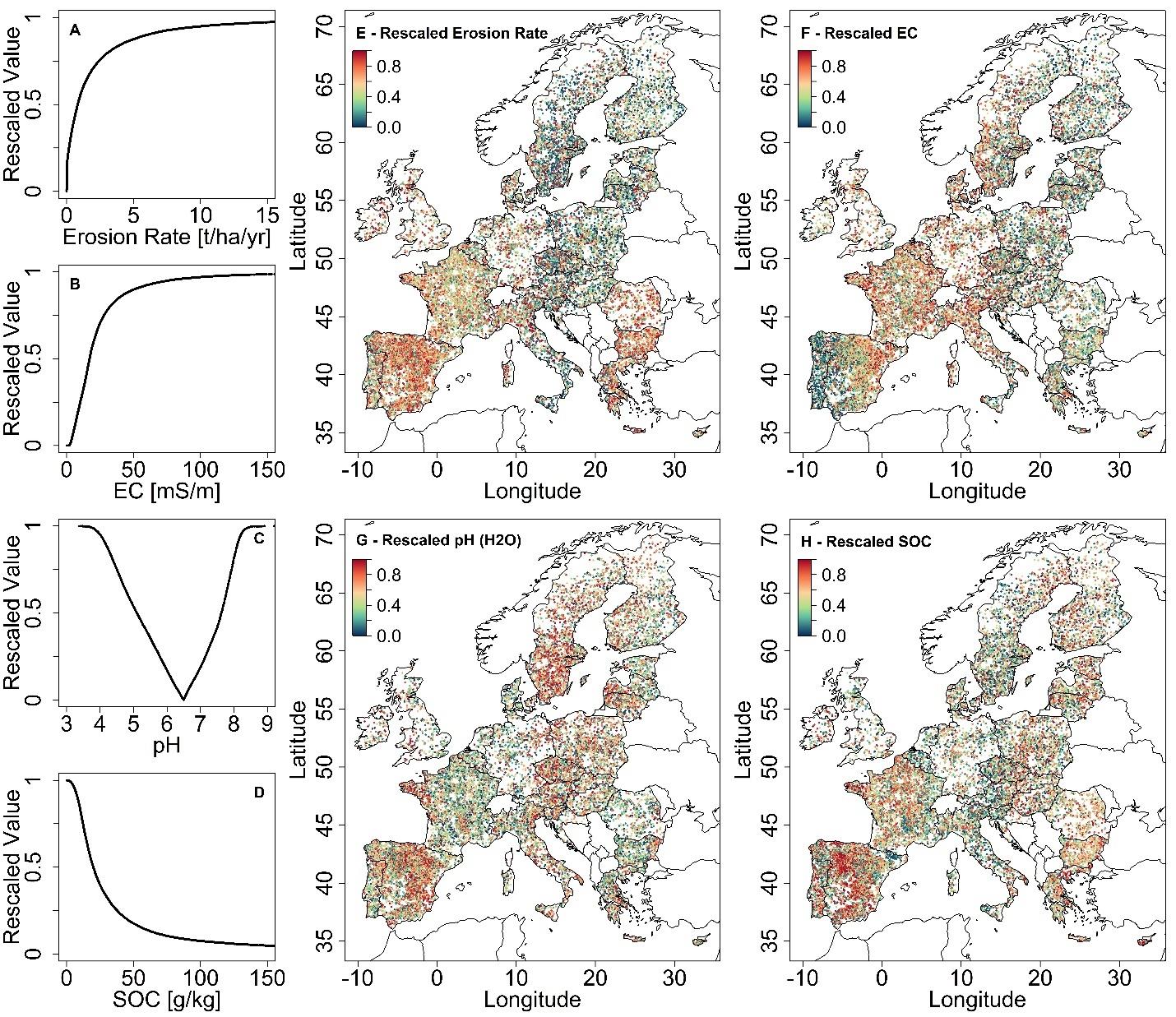


**Supplementary Figure 1.** Relationship between soil health indicators and their rescaled values (CDFs) used in the Soil Degradation Proxy (SDP) development (**A-D**). Spatial distribution of rescaled erosion rate (**E**), rescaled electrical conductivity (**F**), rescaled pH (**G**), and rescaled soil organic carbon (**H**)

**Supplementary Table 1.** Performance metrics of the random forest model across the training (out-of-bag) and external validation datasets. Metrics include the coefficient of determination (R-squared), root mean squared error (RMSE), and mean absolute error (MAE), averaged across the 100 bootstrap models.

| Evaluation set | R-squared | RMSE | MAE |
| --- | --- | --- | --- |
| Training (Out of Bag) | 0.599 | 0.091 | 0.072 |
| Test (External Validation) | 0.602 | 0.091 | 0.072 |


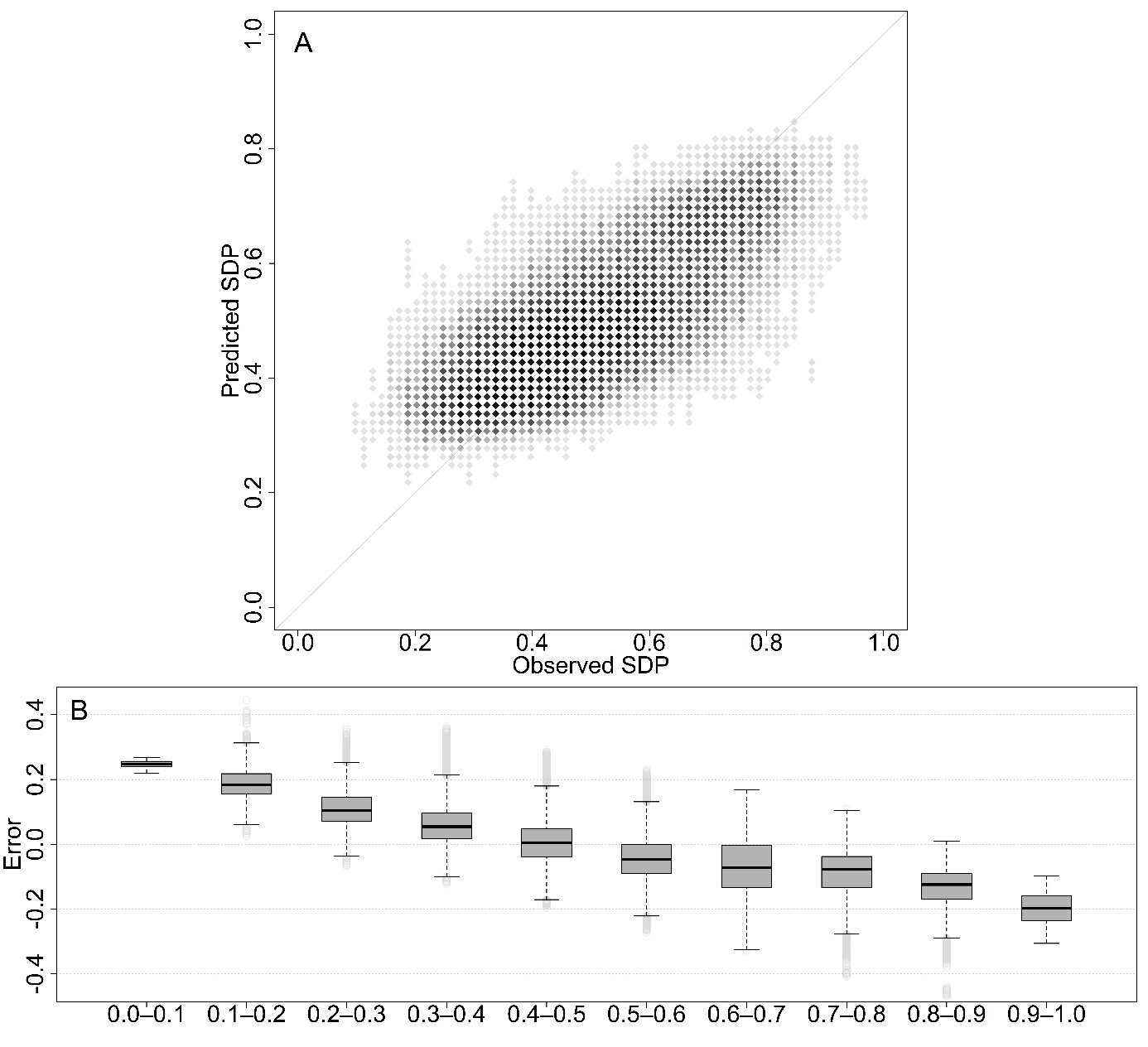


**Supplementary Figure 2.** **(A)** Predicted versus observed Soil Degradation Proxy (SDP) values for the external validation dataset across the 100 bootstrap random forest models. **(B)** Distribution of prediction errors (predicted SDP minus observed SDP) across ten SDP intervals (0-0.1, 0.1-0.2, …, 0.9-1.0), based on the external validation dataset across the 100 bootstrap models. Darker points in the scatterplot of panel A indicate higher density.


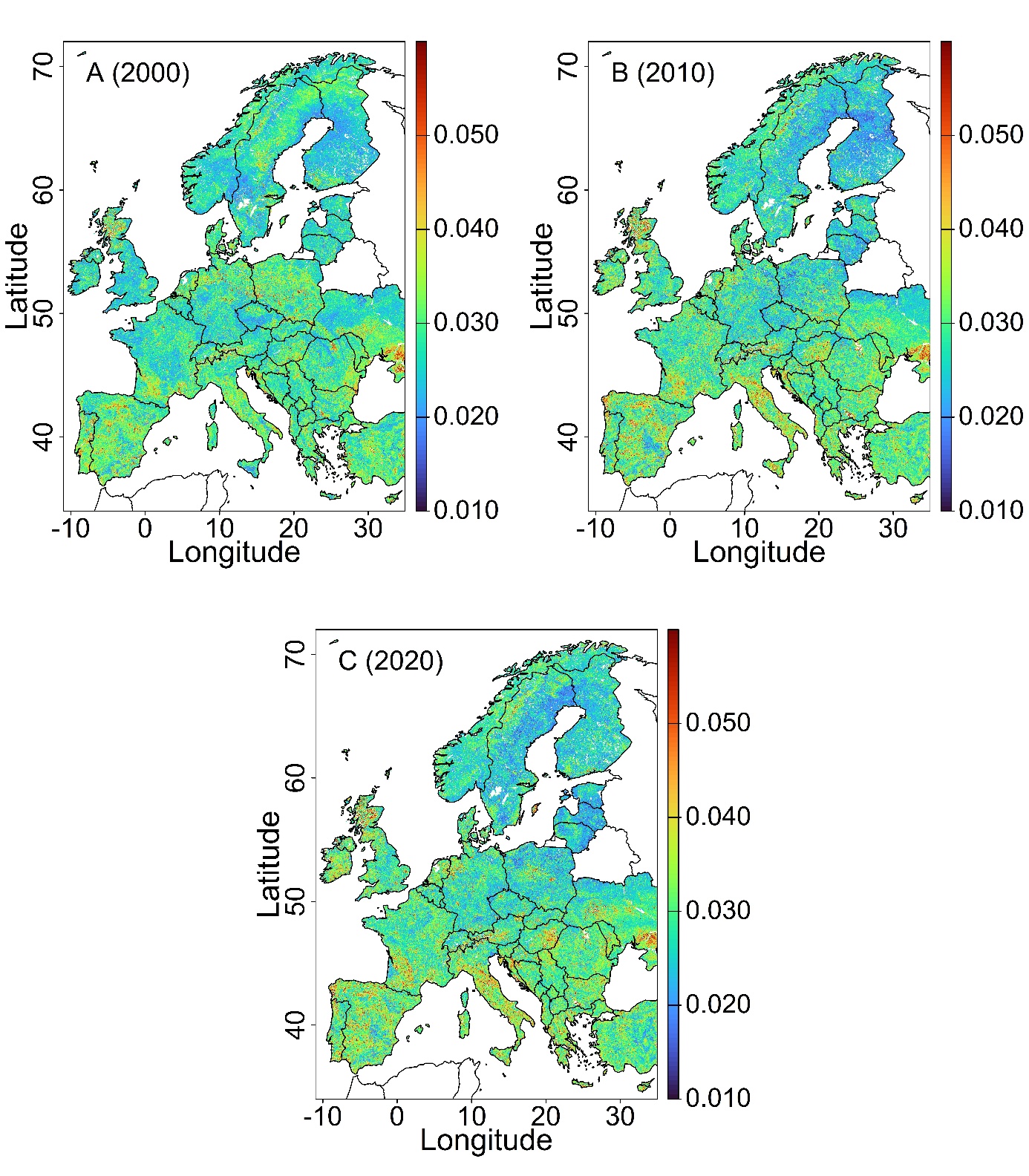


**Supplementary Figure 3.** Uncertainty in predicted SDP for the years **(A)** 2000, **(B)** 2010, and **(C)** 2020, derived from the bootstrap ensemble of 100 random forest models. For each pixel, the uncertainty was estimated as the difference between the 0.95 and 0.05 percentiles of the 100 model predictions. Higher values indicate greater model disagreement and thus higher predictive uncertainty.
