# Supplementary material for: Spatial and temporal assessment of soil degradation risk in Europe
Source: Sci Rep. 2025 Dec 24;15:44636. doi: 10.1038/s41598-025-33318-7 (PMC12749955; doi:10.1038/s41598-025-33318-7)
Supplement: Supplementary file 2 — Supplementary Material 2 [file 41598_2025_33318_MOESM2_ESM.zip › Data/README.pdf]

README

Data Description

This repository contains processed datasets used in the analysis for the study. The datasets are organized into the following files:

- SDP_2015.csv
- SDP_2018.csv
- Components_2015.csv
- Components_2018.csv

Because some of the underlying data originate from licensed datasets, certain modifications were applied to avoid redistribution of restricted spatial information.

Spatial Information in SDP Files

In the original workflow, the datasets contained geographic coordinates (LAT, LNG) corresponding to LUCAS observation points. To comply with data licensing restrictions, these coordinates have been removed.

Instead, the files SDP_2015 and SDP_2018 contain a column named:

LUCAS_Point_ID

Users who wish to reconstruct the spatial coordinates must obtain the official LUCAS dataset from the European Soil Data Centre (ESDAC). The coordinates associated with each LUCAS_Point_ID can be retrieved from the official LUCAS datasets available through ESDAC.

To obtain the coordinates:

1. Request access to the LUCAS soil dataset from the European Soil Data Centre (ESDAC).
2. Retrieve the LAT and LNG values associated with each LUCAS_Point_ID.
3. Join these coordinates with the SDP_2015 and SDP_2018 tables using the LUCAS_Point_ID field.

ESDAC portal:
https://esdac.jrc.ec.europa.eu

Components Files

The files:

- Components_2015.csv
- Components_2018.csv

contain only 300 randomly selected rows from the original component datasets.

These samples are provided solely to illustrate how the analysis scripts operate and to allow users to test the workflow. The full component datasets are not included in this repository in order to comply with licensing restrictions.

Reconstructing the Full Workflow

To fully reproduce the analysis, users will need to retrieve the following variables for LUCAS observation points from ESDAC datasets:

- Soil pH
- Electrical Conductivity (EC)
- Soil Organic Carbon (SOC)
- Erosion Rate derived from the RUSLE soil erosion model

These datasets are available through the European Soil Data Centre (ESDAC).

Once the required datasets are obtained:

1. Extract the variables (pH, EC, SOC, erosion rate) for the relevant LUCAS observation points.
2. Merge them with the LUCAS_Point_ID entries in the SDP files.
3. Replace or extend the example component datasets (Components_2015, Components_2018) with the full datasets.
4. Run the scripts provided in this repository.

Notes

The subset provided in Components_2015 and Components_2018 is not intended for scientific analysis, but only to demonstrate the functionality of the scripts.

For complete reproducibility of the study results, users must obtain the official datasets from the European Soil Data Centre (ESDAC).
